# 242. Evaluation of PBP4 promoter variation and clinical outcomes in patients with *Enterococcus faecalis* bacteremia

**DOI:** 10.1093/ofid/ofad500.315

**Published:** 2023-11-27

**Authors:** Rachel Atterstrom, Truc T Tran, Tariq Nisar, Diana Panesso, Kavindra Singh, Ana Streling, Samie A Rizvi, Shelby R Simar, Stephanie Egge, An Dinh, German Contreras, Blake M Hanson, Marcus Zervos, Lilian M Abbo, Luis Shimose, Samuel A Shelburne, Cesar A Arias, William R Miller

**Affiliations:** Houston Methodist Hospital, Houston, Texas; Houston Methodist Hospital, Houston, Texas; Houston Methodist Research Institute, Houston, Texas; Houston Methodist Research Institute, Houston, Texas; Houston methodist research institute, Houston, Texas; Houston Methodist Research Institute, Houston, Texas; Houston Methodist Hospital, Houston, Texas; University of Texas Health Science Center-Houston, Houston, Texas; Oregon Health Sciences University, Portland, Oregon; Houston Methodist Research Institute, Houston, Texas; University of Texas McGovern Medical School, houston, Texas; The University of Texas Health Science Center, Houston, Texas; Henry Ford Hospital, Detroit, Michigan; University of Miami Miller School of Medicine, Miami Transplant Institute and Jackson Health System, Miami, FL; University of Mississippi Medical Center, Jackson, Mississippi; MD Anderson-University of Texas, Houston,, Texas; Houston Methodist and Weill Cornell Medical College, Houston, TX; Houston Methodist Research Institute, Houston, Texas

## Abstract

**Background:**

Penicillin-binding protein 4 (PBP4) is a low affinity PBP that has been associated with decreased susceptibility to penicillins in *Enterococcus faecalis* (Efs). Changes in the promoter region leading to increased expression of the *pbp4* gene contribute to this phenotype. There is limited data on the clinical outcome of patients infected with these strains in the U.S. We investigated the clinical outcomes of patients with Efs bacteremia stratified by PBP4 promoter type and piperacillin MIC.

**Methods:**

Index Efs bloodstream isolates from 167 patients were selected from the VENOUS cohort (2016-2021). Whole genome sequencing was performed on all isolates and changes in the promoter region (200 bp upstream of the start codon) were identified, using Efs JH2-2 as reference. β-lactam susceptibility (ampicillin, penicillin, and piperacillin [PIP]) was performed on all isolates by broth microdilution. Clinical outcomes (in-hospital mortality, microbiologic failure, and recurrence) were collected on all patients.

**Results:**

The median age for the cohort was 65 years (IQR: 56-72), and 63.5% were male. The median length of hospitalization was 13 days (IQR: 8-22). The duration of bacteremia was 3 days (IQR: 2-4) and 8.98% of patients had prolonged bacteremia (≥ 7 days). Among 167 isolates, 4 major primary promoter variants were identified: reference JH2-2 (n=66), ΔA88 (n=43), A175C (n=35), and insA192 (n=20). All strains were susceptible to ampicillin and penicillin. A PIP MIC ≥ 16 mg/L (non-susceptible) was found in 74.4% of strains with the ΔA88 promoter, as compared to 22.7% of reference, 17.1% of A175C, and 10% of insA192 (p < 0.001). Clinical outcomes of the 167 patients are shown in Table 1. PIP non-susceptibility was associated with immunocompromise state (49.09% vs 23.85%, p=0.001), history of hematological cancer (40% vs 16.51%, p=0.002), and recurrence (7.27% vs 0.92%, p=0.044). There was no statistical difference in mortality or microbiologic failure.

**Figure:**
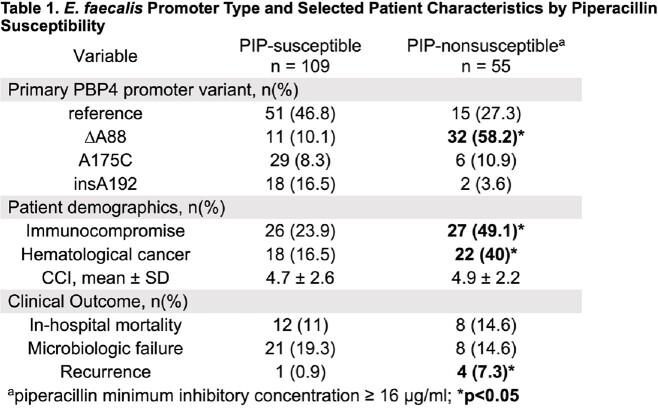

**Conclusion:**

Isolates with ΔA88 PBP4 promoter variants were associated with PIP non-susceptibility. While in-hospital mortality and microbiologic failure were similar between all patients, those who were infected with a PIP non-susceptible strain were more likely to experience a recurrence.

**Disclosures:**

**Marcus Zervos, MD**, Contrafect: Advisor/Consultant|GSK: Grant/Research Support|Johnson and Johnson: Grant/Research Support|Pfizer: Grant/Research Support **Lilian M. Abbo, MD, MBA**, Ferring: Advisor/Consultant|Pfizer: Advisor/Consultant|Regeneron: Grant/Research Support|Shionogi: Advisor/Consultant **William R. Miller, M.D**, Merck: Grant/Research Support|UpToDate: Honoraria

